# A Modified Fokker–Planck Approach for a Complete Description of Vibrational Kinetics in a N_2_ Plasma Chemistry Model

**DOI:** 10.1021/acs.jpca.2c06042

**Published:** 2022-12-29

**Authors:** Margherita Altin, Luca Vialetto, Savino Longo, Pedro Viegas, Paola Diomede

**Affiliations:** † Department of Circular Chemical Engineering, Faculty of Science and Engineering, Maastricht University, PO Box 616, 6200 MDMaastricht, The Netherlands; ‡ DIFFER - Dutch Institute for Fundamental Energy Research, 5612 AJEindhoven, The Netherlands; ¶ Theoretical Electrical Engineering, Faculty of Engineering, Kiel University, Kaiserstraße 2, 24143Kiel, Germany; § Dipartimento di Chimica, Università degli Studi di Bari, 70126Bari, Italy; ∥ Institute for Plasma Science and Technology, National Research Council, Bari Section, Via Amendola 122 /70125, Bari, Italy; ⊥ Department of Physical Electronics, Faculty of Science, Masaryk University, 611 37Brno, Czech Republic; # Instituto de Plasmas e Fusão Nuclear, Instituto Superior Técnico, Universidade de Lisboa, 1049-001Lisboa, Portugal

## Abstract

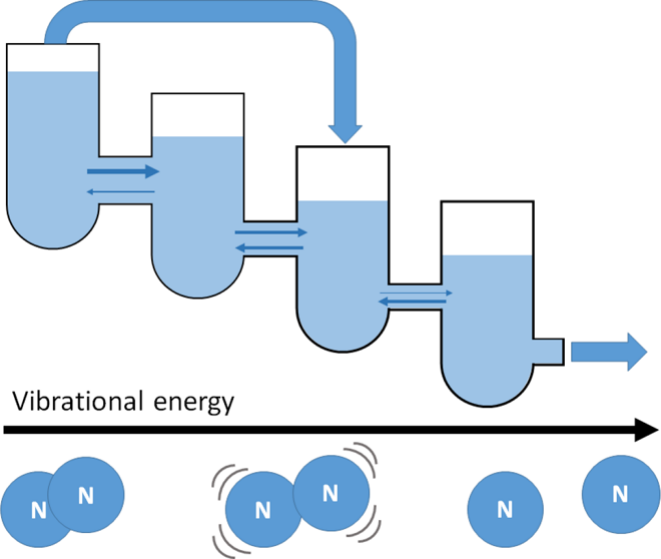

The Fokker–Planck (FP) approach for the description of vibrational kinetics is extended in order to include multiquanta transitions and time dependent solutions. Due to the importance of vibrational ladder climbing for the optimization of plasma-assisted nitrogen fixation, nitrogen is used as a test case with a comprehensive set of elementary processes affecting the vibrational distribution function (VDF). The inclusion of the vibrational energy equation is shown to be the best way to model transient conditions in a plasma reactor using the FP approach. Results are benchmarked against results from the widely employed state-to-state (STS) approach for a wide parameters range. STS and FP solutions agree within ∼10% for the lowest vibrational levels, while time dependent VDFs are in agreement with the STS solution within a ∼ 5% error. Using the FP approach offers the possibility to parametrize drift and diffusion coefficients in energy space as a function of vibrational and gas temperature, providing intuitive and immediate insights into energy transport within the vibrational manifold.

## Introduction

The employment of low temperature plasmas in the field of gas conversion is considered to be a future viable and efficient alternative to conventional methods, which are associated with high energy consumption and production of greenhouse gases.^1−3^ The interest in these techniques is motivated by the high degree of vibrational excitation that can be maintained in plasma discharges, which reduces the energy barrier for molecule dissociation. Several studies have highlighted the role of vibrational excitation in the chemical kinetics of different gases, such as nitrogen,^4^ carbon dioxide,^5^ or methane,^6^ in plasma discharges. The temporal evolution of the population of vibrationally excited states of any molecule is described by the Master Equation (ME),^7^ a system, in the investigated case, containing as many ordinary differential equations (ODEs) as the number of vibrational levels, including hundreds of rate coefficients describing relaxation and excitation processes. Insights into the role of vibrational excitation on gas heating and chemical kinetics in plasmas have been provided using the State-to-State (STS) approach,^8−11^ in which relevant internal energy states are treated as individual pseudospecies, whose evolution is described by rate balance equations. However, the computational efficiency of the STS approach decreases quickly as the number of vibrational states increases. Therefore, applying that method to describe the vibrational kinetics in each of the hundreds of cells of a multidimensional fluid model is unfeasible. 1D fluid modeling of N_2_ plasmas has been conducted in the past to describe nozzle flow expansion^12^ and shock waves,^13^ while Wang et al.^14^ have introduced a 1D fluid model for a quasi-gliding arc, by applying lumping of vibrational levels. Any extension at higher dimensions, without simplifying the full complexity of vibrational kinetics, has not been attempted yet.

Previous works have attempted to increase the efficiency of 0D global models using data-driven approaches that reduce either the number of species involved or the amount of processes considered. In particular, the work by Sahai et al.^15^ introduces an adaptive coarse-grained technique, showing discrepancies in the high energy tail of the vibrational distribution function (VDF) when compared to a full STS solution. Lumping of vibrational levels, as performed by De La Fuente et al.,^16^ allowed prediction of CO_2_ dissociation by considering a fictitious species instead of the complete vibrational manifold; though this provides good agreement with the STS solution in the afterglow region, it assumes a Treanor distribution, which tends to overestimate the population of high energy states. This may cause overheating of the background gas and overpopulation of high energy electrons if a self-consistent solution of the heat equation and the electron Boltzmann equation is employed. Moreover, Berthelot and Bogaerts^17^ have shown good predictions of CO_2_ dissociation fraction by applying a reduction of the chemistry set based on lumping of vibrational levels; the approach shows good agreement with a full model only for pressures higher than 100 mbar and overestimates the tails of the VDF. Kustova et al.^18^ and Kosareva et al.^19^ have worked on multitemperature models applied to CO_2_, showing vibrational temperatures in agreement with the STS solution but pointing out poor agreement in the VDF shape and the possibility of higher computational efficiency only for multicomponent discharges. Finally, the dimensionality of the problem may be reduced employing principal component analysis (PCA), as shown recently by Peerenboom et al.,^20^ without compromising the predictive ability of the model when calculating CO density in a CO_2_ plasma. In order for PCA to work, however, one has to build a training data set, and its accuracy is highly dependent on the choice of scaling parameters and log-transformation that must be set a priori by the user.

As a fast and accurate alternative to the solution of the ME with discrete levels, the present work models the vibrational kinetics as a drift-diffusion problem in a continuous energy space. Commonly known as the Fokker–Planck (FP) approach, this method was first introduced as an analytical method in the 1970s and 1980s^21−24^ and has been recently employed numerically to describe resonant and nonresonant vibrational–vibrational collisions, vibrational–translational relaxation, and vibrational–vibrational collisions between different modes in CO_2_; the approach has been benchmarked by solving the Fokker–Planck equation using a Monte Carlo method^25^ or a flux matching approach.^26,27^ This description is a complete one, in the sense that it is equivalent to a corresponding Master Equation with no level lumping. Details on the modeling of resonant and nonresonant vibrational–vibrational (V–V) relaxation and vibrational–translational (V–T) relaxation in the shape of advective and diffusive transport are summarized in the work by Fridman.^28^ The inclusion of monoquantum electron–vibrational (e–V) processes using the same method has been proposed by Fridman et al.,^23^ but it has not been benchmarked yet. Moreover, the inclusion of multiquanta transitions through the FP approach has yet to be tackled and cannot be ignored, due to the relevance of multiquanta e–V transitions and multiquanta V–T collisions with N in the shaping of the VDF of N_2_.^29^

In addition, recent studies on microwave (MW) discharges have shown that nonequilibrium conditions in the plasma core can be obtained in a pulsed regime.^30^ Modeling those conditions, with the aim of determining their effect on radical production or dissociation, requires a time-resolved knowledge of the molecules VDF, as vibrational kinetics is strongly coupled to both translational degrees of freedom and chemical kinetics. In fact, in the case of N_2_ discharges, V–T relaxation processes due to impact with either N_2_ or N significantly contribute to gas heating;^8^ in turn, the increase in gas temperature leads to changes in the vibrational rate coefficients. Moreover, relevant dissociation and excitation reactions of electronically excited states of N_2_ are also dependent on the population of vibrational states^31,32^

Optimization of nitrogen fixation has received recent attention due to the extensive need of fertilizer production and the environmental impact associated with the currently used Haber–Bosch (H–B) process.^33^ Recently, efforts have been put into the study of microwave plasma reactors using N_2_/O_2_ gas mixtures for the production of nitrogen oxides as a possible alternative to the H–B method.^33,34^ The energy efficiency for the production of NO_*x*_ using atmospheric nitrogen as a precursor is hindered by the stability of the triple bond in the N_2_ molecule. However, the thermodynamic nonequilibrium conditions maintained in MW reactors, where vibrational levels of N_2_ are populated by collisions with electrons, can lower the dissociation energy barrier, providing room for optimization. Motivated by this, N_2_ is used in this work as a test-case for the application of the FP approach.

In the present work, the validity and limitations of the FP approach for a time-resolved description of the vibrational kinetics of N_2_ are studied. First, the calculation of advection and diffusion coefficients in vibrational energy space is presented, along with the modified form of the FP equation, due to the introduction of multiquanta collisions. Then, several methods for the calculation of a time-resolved, self-consistent vibrational temperature are introduced. Benchmarking against STS solutions is then conducted at different degrees of complexity, in order to highlight the limits of validity of the extended FP method in the parameters space.

## Methods

### State-to-State Model

The STS model considers only the electronic ground state of N_2_ with 46 vibrational levels, following the calculations done using an anharmonic Morse oscillator potential with vibrational quantum *w*_*e*_ = 2358.57 cm^–1^ and anharmonicity factor *x*_*e*_ = 6.073, already validated by previous works.^4,8,35,36^ It must be noted that a vibrational manifold with 61 vibrational levels has been proposed in recent years for N_2_;^37^ 46 levels were chosen instead due to the more extensive literature providing rate coefficients of such system. The full list of the processes included to describe vibrational kinetics can be found in Table 1 and will be included gradually in order to allow a thorough comparison with the FP method. Note that, with respect to the state-of-the-art vibrational kinetics scheme proposed by Guerra et al.,^29^ collisions between molecules excited to any vibrational level *v* and molecules excited to the vibrational level *i* (V–V_*i*_ processes) with *i* > 2 are not included. This is due to their progressively less relevant contribution to the VDF, at values of electron density commonly found in MW nitrogen discharges,^10,30^ which will be shown in the Results.

**Table 1:** Reactions Scheme for the Description of the Vibrational Kinetics in N_2_^a^

process name	reaction
e–V	e + N_2_(*v* = *n*) ↔ e + N_2_(*v* = *m*);*n* = 0–45, 0 < (*m* – *n*) ≤ 10
V–V_1_	N_2_(*v* = *n*) + N_2_(*v* = 1) ↔ N_2_(*v* = *n* + 1) + N_2_(*v* = 0)
V–V_2_	N_2_(*v* = *n*) + N_2_(*v* = 2) ↔ N_2_(*v* = *n* + 1) + N_2_(*v* = 1)
V–V_*n*_	N_2_(*v* = *n*) + N_2_(*v* = *n*) ↔ N_2_(*v* = *n* + 1) + N_2_(*v* = *n* – 1)
V–T	N_2_(*v* = *n*) + N_2_ ↔ N_2_(*v* = *n* – 1) + N_2_
	N_2_(*v* = *n*) + N ↔ N_2_(*v* = *m*) + N, max[0, *n* – 5] ≤ *m* < *n*
e–V (diss.)	e + *N*_2_(*v*) → e + N + N, *v* > 35
V–V_1_ (diss.)	N_2_(*v* = 45) + N_2_(*v* = 1) → N + N + N_2_(*v* = 0)
V–V_2_ (diss.)	N_2_(*v* = 45) + N_2_(*v* = 2) → N + N + N_2_(*v* = 1)
V–V_*n*_ (diss.)	N_2_(*v* = 45) + N_2_(*v* = 45) → N + N + N_2_(*v* = 44)
V–V (diss.)	N_2_(*v* = *n*) + N_2_(*v* = *m*) → N + N + N_2_(*v* = 0),10 ≤ n, *m* ≤ 25
V–T (diss.)	N_2_(*v* = 45) + N_2_ → N + N + N_2_
	*N*_2_(*v*) + N → N + N + N, *v* > 40
recombination	N + N + N_2_ → N_2_(*v* = 0) + N_2_

a List of processes taken from ref (29).

In the presence of dissociation, in order to close the system, the recombination reaction
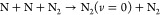
with rate coefficient
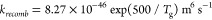
1is considered, with *T*_g_ being the gas temperature. Note that this reaction should normally lead to N_2_(B) formation, instead of N_2_(*v* = 0),^29^ but detailed chemical kinetics is beyond the scope of this work. Moreover, recombination to higher vibrational levels is neglected, due to their absence in the kinetics scheme chosen as reference for this benchmark study;^29^ the effect of such processes would lead to changes in the shape of the tail of the distribution, as their population would be determined by the thermodynamic equilibrium between the density of atomic and molecular nitrogen.

The rate coefficients for V–V_1_, V–V_2_, V–V_*n*_, and V–T collisions are calculated following the work from Adamovich et al.,^35,38^ where they are derived according to the forced harmonic oscillator (FHO) theory, which provides very good agreement with quantum classical calculations^39^ even at higher temperature and is therefore preferred for the simulations of high temperature plasmas.^37^ In contrast, the parametrization presented by Guerra et al.,^29^ based on first-order perturbation theory, is not suitable for high collision energies and deviates from semiclassical calculations^39^ for temperatures above 1000 K.

The rate coefficients for V–T collisions with atomic nitrogen are calculated as suggested in a recent review by Guerra et al.^29^ Finally, rate coefficients for electron impact vibrational excitation (e–V) are calculated as a function of electron mean energy by solving the Boltzmann equation for electrons at different values of reduced electric field using the two-term solver BOLSIG+,^40^ with cross sections obtained from the IST-Lisbon database in LXCAT,^41^ which includes transitions involving the exchange of up to 10 vibrational quanta.

### Fokker–Planck Approach

The detailed derivation of the FP equation from the ME for vibrational kinetics can be found in the book by Van Kampen^7^ or in the book by Bibermann et al.^42^ and is summarized in the work by Viegas et al.^27^ Here, the formulas and notation are briefly introduced.

The Fokker–Planck equation has the form
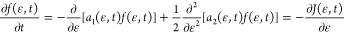
2*f*(ϵ) is a double differentiable function chosen so that its value calculated at energy ϵ_*v*_, which is the energy of the vibrational level *v*, is equal to *n*(ϵ_*v*_), where *n* denotes the discrete vibrational distribution function. This approach allows one to intuitively pass from the FP solution to the VDF, but normalization of *f* has to be enforced and will be described in the section devoted to the integration of the FP equation.

Equation 2 has the same form of a continuity equation without the source term and it can be obtained by truncating the Kramers–Moyal expansion of the Master Equation describing the evolution of *f*.^43^ The addition of a source term is otherwise necessary to describe processes for which such assumption is not true and will be discussed later. *a*_1_(ϵ) and *a*_2_(ϵ) in eq 2 are the first and the second moment of the transition probability. *J*(ϵ) is the flux of energy in the vibrational energy space and can be expressed in terms of drift (*A*(ϵ)) and diffusion (*B*(ϵ)) coefficients as
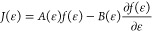
3
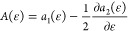
4
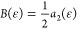
5Note that the explicit dependence on time *t* was dropped for the sake of conciseness of the formulas. This approach will be adopted from now on, unless a derivative with respect to time is present. The coefficients *a*_1_(ϵ) and *a*_2_(ϵ) are defined as
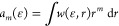
6where *w*(ϵ, *r*) is the probability of a transition from energy ϵ to energy ϵ + *r*, with *r* being the energy jump; *a*_*m*_(ϵ) coefficients are calculated as a sum of the contributions from all the considered processes:
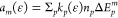
7where *k*_*p*_(ϵ) is the rate coefficient of process *p*, Δ*E*_*p*_ is the variation in vibrational energy and *n*_*p*_ is the number density of the collision partner.

Due to the underlying assumption of small energy jumps, the FP approach as described by eq 2 can efficiently include only monoquantum transitions: attempts to describe 2-quanta processes with the flux formulation provided results in disagreement with the STS method. Hence, the inclusion of multiquanta proccesses is achieved by adding to the FP eq (eq 2) a source term *S*(ϵ)
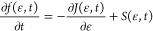
8where
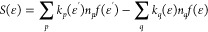
9where indexes *p* and *q* run over all processes causing either the creation or the destruction, respectively, of molecules at vibrational energy ϵ and *n*_*p*,*q*_ are the number densities of the collision partners. This approach resembles the one adopted by Braglia to include inelastic collisions in the description of the time evolution of the electron distribution function.^43^ Its application to the description of vibrational kinetics has never been attempted.

The expressions used to compute drift and diffusion coefficients for the monoquantum processes and the source terms for the multiquanta collisions included in the vibrational kinetics are introduced in the following sections.

#### V–V_1_ and V–V_2_

A molecule undergoing a nonresonant V–V collision gains one quantum of energy by colliding with a target molecule excited to the first vibrational level. Considering also the reverse reaction, having as collision partner a molecule on the ground state, the drift and diffusion coefficients for V–V_1_ are calculated as
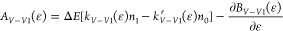
10
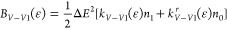
11where *n*_0_ and *n*_1_ are the number densities of the vibrational ground state and the first vibrationally excited state respectively and the superscript *r* identifies reverse reactions, according to the convention in Table 1. Δ*E* is the energy exchange involved in the process and depends on ϵ; this is not explicitly indicated in the formulas for A and B, in order to slim down the notation. It is defined as Δ*E*(ϵ) = ϵ_*v*+1_ – ϵ_*v*_ if ϵ ∈ [ϵ_*v*_; ϵ_*v*+1_), where *v* identifies the vibrational level.

Note that, assuming steady state conditions (d*J*(ϵ)/dϵ = 0) and absence of dissociation (*J*(ϵ_*diss*_) = *J* = 0, where ϵ_*diss*_ is the dissociation energy), eq 2 can be rewritten as
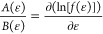
12This relation was used instead of eq 10 to calculate *A*_*V*–*V*1_(ϵ) from *B*_*V*–*V*1_(ϵ) by Viegas et al.^27^

Equations 10 and 11 are also used to calculate drift and diffusion coefficients in energy space for V–V_2_ processes, if *n*_0_ and *n*_1_ are replaced with *n*_1_ and *n*_2_, respectively.

Both V–V_1_ and V–V_2_ collisions can induce dissociation, as listed in Table 1. The inclusion of dissociation due to monoquantum processes is discussed further on. It will be shown later in this work that nonresonant V–V processes through collisions with molecules with higher vibrational energy, which typically have lower population density, are not necessary for an accurate description of the vibrational kinetics.

#### V–T

V–T collisions allow the transfer of energy from vibrational to translational degrees of freedom and vice versa. In such processes, a molecule excited at level *v* = *n* loses (gains) one quantum of energy, jumping to *v* = *n*–1 (*v* = *n* + 1) by colliding with any gas molecule. *A*_*V*–*T*_(ϵ) and *B*_*V*–*T*_(ϵ) are calculated as
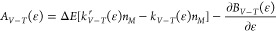
13
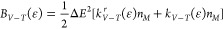
14where *n*_*M*_ is the number density of the collision partner; in the case of V–T, transitions the reverse process identified with the superscript *r* corresponds to the transition from *v* = *n* to *v* = *n* + 1. The definitions given in eq 13 and eq 14 are valid both for monoquantum collisions with molecular and atomic nitrogen.

V–T collisions between N_2_ and molecules excited to the last vibrational level cause dissociation; their inclusion in the FP framework is discussed further on.

#### Multiquanta V–T with N

Losses and gains of vibrational quanta through collisions with atomic nitrogen are deemed as a relevant channel for loss of vibrational energy to translational degrees of freedom.^29^ Multiquanta exchanges can be modeled by introducing a source term *S*_*V*–*T*,*N*_(ϵ), which is calculated for discrete vibrational levels (identified by index *l*) as
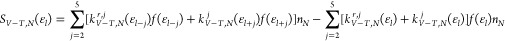
15where *j* is the width of the jump expressed in number of vibrational levels and *n*_*N*_ is the number density of atomic nitrogen, which is fixed in this work. In the first sum on the right-hand side it is required that *j* – *l* > 0 and *j* + *l* ≤ 45. Similarly, the second term of the second sum is different from zero only if the jump in energy space leads to *l* – *j* > 0. The first term, instead, may contain reverse V–T collisions leading to levels above the last vibrational level: these collisions are considered to cause dissociation. The number of quanta exchanged are considered up to a maximum of 5 and their rate coefficients are taken from a recent review by Guerra et al.^29^ Moreover, monoquantum exchanges can be easily modeled using eqs 13 and 14 and therefore are not included in eq 15.

#### V–V_*n*_

Quasiresonant V–V processes proceed through a collision between two molecules on the same vibrational level, leading thus to a nonlinear dependence on the VDF.

A workaround suggested by Viegas et al.^27^ consists in defining the ratio between *A*_*V*–*Vn*_ and *B*_*V*–*Vn*_, based on the expected equilibrium VDF in the absence of other processes, i.e., a Treanor distribution. Such a solution, however, although being a possible one, is not unique.^28^ This work will therefore proceed in deriving drift and diffusion coefficients from the definition. As presented above, eq 7 provides an explicit way to calculate the moments of transition probability evaluated at vibrational energy ϵ, therefore including only rate coefficients calculated at said ϵ. Hence, the calculation of a specific *a*_*m*_(ϵ_*v*_) (where ϵ_*v*_ is the vibrational energy of level *v*), should only include V–V_*n*_ processes involving N_2_(*v* = *n*) as a reactant, that is
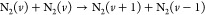
16
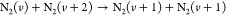
17
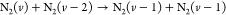
18In the following, the second reactants in reactions (16), (17) and (18) will be considered as targets, exactly as *n*_0_ and *n*_1_ in the derivation for V–V_1_ processes. Such assumption is what allows the description of the inherently nonlinear V–V_*n*_ processes in the form of a drift and a diffusion term, instead of the expression derived by Fridman et al.^28^

For the sake of the derivation, the two reverse processes (eqs 17 and 18) are identified with two different names, respectively *k*_*R*2_(ϵ) and *k*_*R*_(ϵ), while for the sake of notation, the rate coefficient for the forward process is referred to as *k*_*F*_(ϵ). Accordingly, the first two moments of the transition probability are
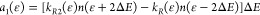
19
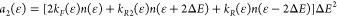
20The absence of the process in eq 16 from the expression of the first moment is due to the fact that it appears twice in eq 7, once with a variation in vibrational energy equal to Δ*E* and once with −Δ*E*; the two contributions therefore cancel out.

Considering the definition of A(ϵ) (eq 4) and substituting *a*_1_ and *a*_2_:
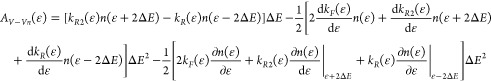
21while *B*_*V*–*Vn*_(ϵ) is calculated simply as *a*_2_(ϵ)/2. The terms calculated at (ϵ+2Δ*E*) or (ϵ–2Δ*E*) are considered zero if (ϵ ± 2Δ*E*) ∉ [0, ϵ_*diss*_], where ϵ_*diss*_ is the dissociation energy.

Equation 21 contains terms with the derivative of the VDF with respect to energy; calculating those terms at every iteration leads to an increase of computational time of ∼20%. As a workaround to this, one can consider all the terms containing a derivative as being calculated in ϵ
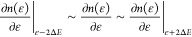
22and write the drift coefficient as
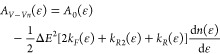
23where *A*_0_(ϵ) contains the first two terms on the RHS of eq 21.

By plugging *A*_*V*–*Vn*_(ϵ) and *B*_*V*–*Vn*_(ϵ) in the definition of flux in energy space (eq 3):
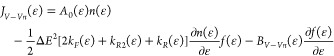
24
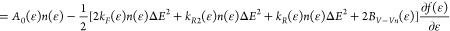
25where the second passage involves the fact that . In this way, the last term in eq 21 is included in the definition of *B*_*V*–*Vn*_(ϵ), avoiding the explicit calculation of the derivative every time the coefficients are recalculated. More about what the approximation in eq 22 entails is discussed in the Results. Similarly to what was said for previous processes, V–V_*n*_ involving the last vibrational level can cause dissociation.

Last, the previously described V–V_1_ and V–V_2_ processes coincide with V–V_*n*_ collisions with *n* = 1 and *n* = 2. In order not to double-count such processes, *J*_*V*–*Vn*_(ϵ) is considered 0 in the vibrational energy interval [0, ϵ_3_).

#### Multiquanta V–V

Table 1 contains V–V collisions leading to dissociation, hence involving the exchange of more than a single quantum of energy. They are modeled similarly to what was already introduced for multiquanta V–T collisions with atomic nitrogen, i.e., with an additional sink term:
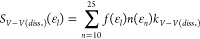
26where *k*_*V*–*V*(*diss*.)_ is the rate coefficient for V–V driven dissociation and does not depend on *l*.^29^

#### e–V

Electrons are responsible for the initial pumping of energy into the vibrational manifold. This occurs through multiquanta transitions, which violate the small energy jumps assumption on which the Fokker–Planck approach is based. As previously explained, the introduction of a source term allows the inclusion of such processes in the FP framework. The source term due to e–V collisions is calculated as
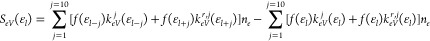
27where the notation is analogous to the one used for multiquanta V–T on N atoms, with *n*_*e*_ being the electron number density, which is fixed in this work. Also in this case, dissociation is included by admitting the first term in the second sum to include also e–V collisions reaching ϵ_*diss*_.

*S*_*eV*_ is calculated for discrete vibrational levels and is then linearly interpolated on the nodal points of the grid to find the value at ϵ. A possible formulation of e–V collisions using FP formalism has been proposed by Macheret et al.^22^ and summarized by Fridman^23,28^ In the latter, a diffusion coefficient in energy space is introduced as *D*_*eV*_ so that the flux due to monoquantum e–V collisions assumes the form
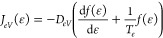
28

*T*_*e*_ is the electron temperature, which is fixed in this work. This expression can be obtained by assuming the EEDF as a Boltzmann distribution, which is seldom a realistic case, and *D*_*eV*_ as a constant value, independent of vibrational energy. In order to generalize the approach, this work uses instead *A*_*eV*_(ϵ) and *B*_*eV*_(ϵ) calculated following the same formulation given for V–T processes:
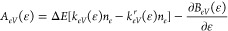
29
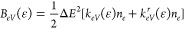
30where *n*_*e*_ is the electron density and *k*_*eV*_(ϵ) is the rate coefficient only for monoquantum processes.

In this work, this approach is compared to the inclusion of an *S*_*eV*_(ϵ) term in the FP equation in the simple case where only monoquantum energy transfers are allowed.

### Self-Consistent Vibrational Temperature Calculation

With the presence of e–V processes, it is possible to obtain a self-consistent value of vibrational temperature or, in other words, a self-consistent population for N_2_(*v* = 0) (*n*_0_) and N_2_(*v* = 1) (*n*_1_). Since *n*_0_ and *n*_1_ are then used in the definition of *A*_*V*–*V*1_(ϵ) and *B*_*V*–*V*1_(ϵ), an accurate determination of the vibrational temperature (*T*_*v*_) is important in order to obtain the correct VDF with the FP approach.

In this work, three different approaches to the calculation of vibrational temperature are presented and compared in terms of accuracy and computational efficiency:

i Fokker–Planck equation coupled with the solution of the vibrational energy equation;
ii Fokker–Planck equation coupled with a reduced STS scheme;
iii Fokker–Planck equation alone.

As suggested by Rusanov et al.,^44^ a possible way to model the time evolution of vibrational temperature is defining an energy balance equation for the mean vibrational energy (⟨ϵ_*v*_⟩) (case i):
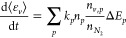
31where *k*_*p*_ is the rate coefficient for the process *p*, *n*_*p*_ is the density of the collision partner, Δ*E*_*p*_ is the energy exchange involved in the process, *n*_*v*,*p*_ is the population of the vibrational level *v* (obtained from the solution of the FP equation) involved in the collision *p* and  is the density of nitrogen molecules. In this work, the processes included in eq 31 are vibrational–translational collisions with N_2_ and N, quasiresonant and nonresonant vibrational–vibrational collisions, e–V, and supereleastic collisions with electrons. In the presence of an extended chemistry, where N_2_ vibrational states interact with chemical species, such reactions should also be included in eq 31.

The relation between vibrational temperature *T*_*v*_ and mean vibrational energy ϵ_*v*_ is given, as reported by Fridman,^23,28^ by Planck’s formula:
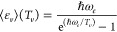
32Equation 32 provides the mean vibrational energy if a discrete Boltzmann distribution is assumed, ignoring the anharmonicity of the vibrational energy levels.

The population of the first vibrational level is consequently calculated from *n*_0_ inverting the conventional definition of vibrational temperature:
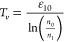
33where ϵ_10_ is the energy difference between the first vibrational level and the ground state. The obtained value of *n*_1_ is then used to calculate the drift and diffusion coefficients for V–V_1_ processes as defined in eq 10 and 11.

As an alternative method (case ii), N_2_(*v* = 0) and N_2_(*v* = 1) populations can be calculated with a STS approach, including only relevant e–V, V–V_1_, V–T, and V–V_*n*_ reactions, using as input for the populations of vibrational levels the ones obtained from the solution of the FP equation; in the present case, the reactions in the reduced STS model are listed in Table 2. The values of *n*_0_ and *n*_1_ obtained from such solution are then used as input for eqs 10 and 11. Those processes are the ones with the highest reaction rate, based on the results in this work. Vibrational temperature is then calculated using eq 33. This approach offers the advantage of not needing any assumptions when calculating *T*_*v*_.

**Table 2:** Reaction Scheme Considered in the Reduced STS Model

process name	reaction
e–V	e + N_2_(*v* = 0) ↔ e + N_2_(*v* = *m*); 0 < *m* ≤ 10
	e + N_2_(*v* = 1) ↔ e + N_2_(*v* = *m*); 1 < *m* ≤ 11
V–V_1_	N_2_(*v* = *n*) + N_2_(*v* = 1) ↔ N_2_(*v* = *n* + 1) + N_2_(*v* = 0); *n* = 2–10
V–V_*n*_	N_2_(*v* = 1) + N_2_(*v* = 1) ↔ N_2_(*v* = 0) + N_2_(*v* = 2)
	N_2_(*v* = 2) + N_2_(*v* = 2) ↔ N_2_(*v* = 1) + N_2_(*v* = 3)
V–T	N_2_(*v* = 1) + N_2_ ↔ N_2_(*v* = 0) + N_2_
	N_2_(*v* = 2) + N_2_ ↔ N_2_(*v* = 1) + N_2_

Lastly, for case iii, *T*_*v*_ is simply obtained by the evolution of the VDF, by letting *n*_0_ and *n*_1_ evolve according to the calculated drift and diffusion coefficients.

### Integration of the Fokker–Planck Equation

Equation 8 is solved numerically using a code developed by the authors, which employs the control volume technique described by Patankar,^45^ implementing the tridiagonal matrix algorithm (TDMA) to invert the resulting tridiagonal matrix, also described by Patankar in pp 52–54 of the latter reference.

The solution domain extends from 0 eV to the N_2_ dissociation energy (10.6 eV) and is divided into 1000 control volumes, which allows the energy width of the cell to be much smaller than the energy jumps between levels, while maintaining accuracy of the solution. Higher numbers of control volumes have been tried, obtaining convergence of the solution using 10000 cells. Levels between 4 and 40 are not significantly affected by such change, while the error on the remainder is within 5%.

*A* coefficients, accounting for the advective motion, are defined at the interface between two volumes, while *B* coefficients are defined on nodal points. Due to the dominance of diffusive motion and the consequent low value of the Peclet number, a central difference scheme has been employed as interpolation scheme.^45^

The boundary conditions for the solution are nonhomogeneous Neumann from the flux definition in eq 3, expressed as
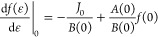
34
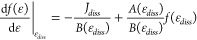
35where *J*_0_ is the flux of particles entering the domain due to recombination leading to the formation of N_2_ in the vibrational ground state (last reaction in Table 1) and is calculated as
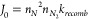
36*J*_*diss*_ in eq 35 is the flux in energy space accounting for particles leaving the domain due to dissociation and is calculated as
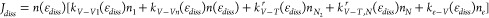
37Strictly speaking, the only contributions to *J*_*diss*_ are the ones causing a movement in energy space from the cell close to the boundary to the outside of the domain. Hence, only monoquantum exchanges can be considered part of it. Any contribution to dissociation involving multiquanta transitions from vibrational levels different from the last one should be taken into account by means of a loss term, just as defined above, and they are not considered part of the dissociation flux at the boundary.

The normalization of the distribution is kept in check by multiplying at every iteration the populations by a factor:
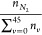
38where  is calculated as
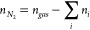
39where the gas density (*n*_*gas*_) is defined by the ideal gas law at *p* = 100 mbar and *T*_g_ and the sum runs over all other species in the model.

Moreover, time integration is performed using a fully implicit scheme, and the time step Δ*t* is chosen so as to satisfy
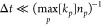
40i.e., the time step is much lower than the characteristic time of the fastest reaction and is kept fixed throughout the simulation; convergence of the solutions is obtained already at ∼20% of the upper limit. In the conditions investigated in this manuscript, the fastest processes are e–V and V–T collisions (for the high energy end of the VDF); since their rate depends on electron and gas density, which are kept fixed, the upper limit in eq 40 can be calculated a priori. Typically, their time scale is ∼0.1 μs, making the time step typically around 0.01 μs.

## Results and Discussion

A summary of the different test cases used for the benchmarking of the FP approach can be found in Table 3.

**Table 3:** Summary of the Different Cases Presented in the Results

section	vibrational kinetics	self-consistent *T*_*v*_	time evolution	dissociation/recombination
V–V_1_	V–V_1_	no	no	no
V–T	V–T	no	no	no
V–T	V–V_1_, V–T	no	no	no
V–V_*n*_	V–V_1_, V–T, V–V_*n*_	no	no	no
e–V and vibrational temperature	V–V_1_, V–T,V–V_*n*_, e–V	yes, with methods i–iii	yes	no
V–V_2_	V–V_1_, V–T, V–V_*n*_,e–V, V–V_2_	yes, with method i	no	no
collisions with atomic nitrogen	V–V_1_, V–T, V–V_*n*_,e–V, V–V_2_, V–T,N	yes, with method i	no	no
dissociation mechanisms	Table 1	yes, with method i	no	yes
time evolution	Table 1	yes, with method i	yes	yes

For the purpose of benchmarking, STS results will be presented with a single process (V–V_1_ or V–T), in which case the populations of the vibrational ground state and of the first vibrationally excited state are fixed, and with multiple processes, without fixing *T*_*v*_. Those case studies where more than one process is included (for example, V–V_1_ and V–T, or V–V_1_, V–T, and V–V_*n*_), and the complete set of e–V is not included, are obtained by introducing one quantum e–V and superelastic collisions exclusively between the vibrational ground state and the first excited state. Different values of vibrational temperature are obtained by changing the electron mean energy and *T*_*e*_. This was performed in order to obtain conservation of the total number of particles in the STS solution. The values of *n*_0_ and *n*_1_ obtained from the STS solution are then kept fixed in the FP solution, effectively fixing the vibrational temperature. This was not necessary for the kinetic scheme including only V–V_1_ or V–T, as the resulting theoretical distribution is known and it is trivial to calculate the correct values of *n*_0_ and *n*_1_, taking into account the normalization of the distribution.

The integration of the system of ODEs is done with RADAU5,^46^ a Runge–Kutta solver of order 5,^47^ but simulations employing LSODA^48^ and the simpler forward method are also carried out, in order to provide a more complete benchmark of computational efficiency.

In order to obtain results that can be compared in terms of CPU time, STS and FP simulations were carried out by fixing the physical time limit to 10 ms. All other simulations, which serve as a benchmark of the FP approach, without actual comparison of computational efficiency, are stopped when the following convergence criterion is matched:
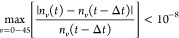
41where *n*_*v*_(*t*) is the population of the vibrational level *v* and *n*_*v*_(*t* – Δ*t*) is the value of the same population at the previous time step. The same convergence criterion stands for the FP simulation, using *f*(ϵ_*v*_) instead of *n*_*v*_.

### V–V_1_ Processes

**Figure 1:**
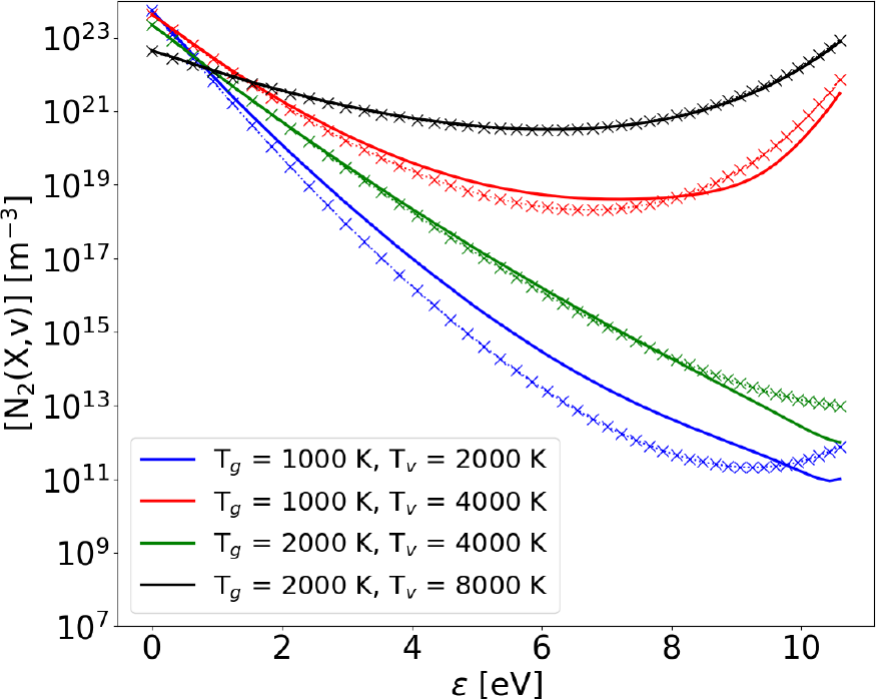
Vibrational distribution functions obtained with the FP approach (solid lines) compared to the ones obtained with the STS method (crosses) and the theoretical Treanor distribution (dots) including only V–V_1_ processes (with no dissociation) at different values of gas and vibrational temperature.

The inclusion of only V–V_1_ processes in the vibrational kinetics, with the absence of dissociative processes, leads to a Treanor distribution:^49^
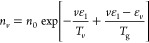
42Figure 1 shows the comparison between the steady state VDF obtained with the Fokker–Planck method and the one obtained through a STS approach (which provides the theoretical Treanor distribution), at different input values of vibrational and gas temperature, choosing values that could realistically be present in a MW reactor.^50^ The effect of changes of both temperature values is clearly visible. In fact, for the Fokker–Planck equation to have a Treanor solution at vibrational temperature *T*_*v*_ and gas temperature *T*_g_, the value of *A*_*V*–*V*1_(ϵ) and *B*_*V*–*V*1_(ϵ) have to satisfy the following condition:
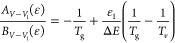
43This relation can be obtained by noticing that in the absence of processes causing dissociation, *J* = 0, and therefore, eq 3 becomes
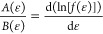
44as already pointed out by Viegas et al.^27^

The actual value of *A*/*B* has been calculated explicitly from eq 10 and eq 11, by noticing that rate coefficients for forward and reverse reactions are linked by detailed balance. The obtained analytical expression for *A*/*B* has been compared to the expected one (eq 43), yielding the conditions necessary for a matching solution:
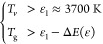
45While the first condition on *T*_*v*_ is easily translated into a single numerical value, the one on gas temperature depends on the energy level, with ϵ_1_ – Δ*E*(ϵ) increasing in value toward the tail of the distribution, where ≈1700 K is reached. Figure 1 shows an improvement of the agreement between STS and FP solution as *T*_*v*_ and *T*_g_ are increased.

### V–T Relaxation

In a system where only V–T processes are included, a Boltzmann distribution at the gas temperature *T*_g_ is reached by the VDF. The ratio between drift and diffusion coefficients required to obtain that solution is
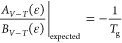
46

**Figure 2:**
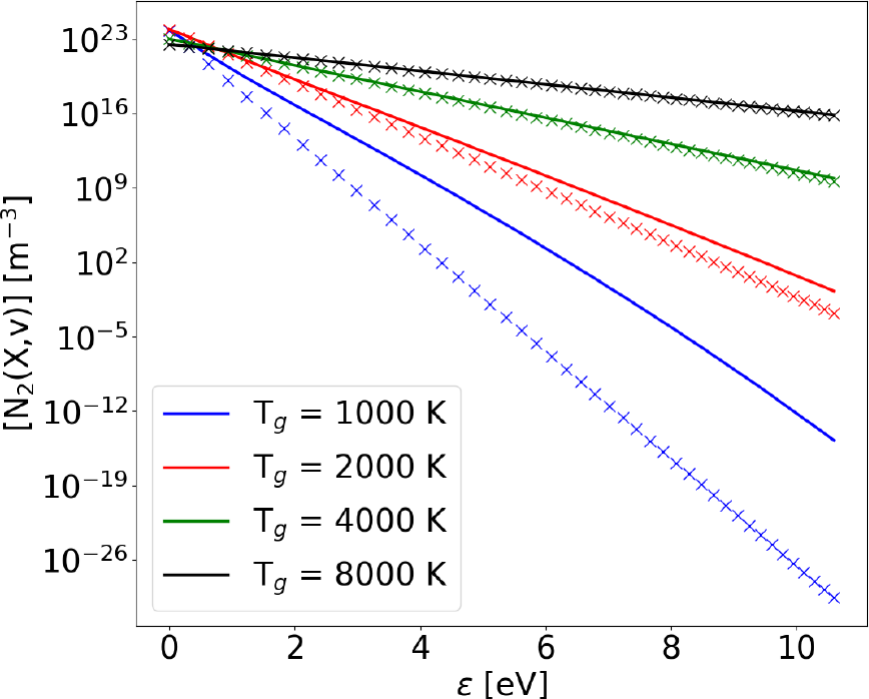
Vibrational distribution functions obtained with the FP approach (solid lines) compared to the ones obtained with the STS method (crosses) including only V–T processes (with no dissociation) at different values of gas temperature. In this case, the vibrational temperature has the same value as the gas temperature.

By applying the same procedure as for V–V_1_, the limiting condition in this case is
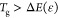
47where Δ*E* varies from 0.164 eV (at the high energy end of the distribution) to 0.314 eV (at the lower energy end of the distribution). A good agreement can thus be found at all levels if *T*_g_ > 3700 K, as demonstrated by Figure 2, where the steady state VDF in the presence of only V–T collisions is calculated using the FP approach and compared to the STS solution, which is a theoretical Boltzmann with *T*_*v*_ = *T*_g_.

However, when a complete kinetic scheme is implemented, V–T relaxation is expected to be dominant at the high energy end of the distribution,^28^ where Δ*E*(ϵ) is lower, allowing also lower values of *T*_g_.

**Figure 3:**
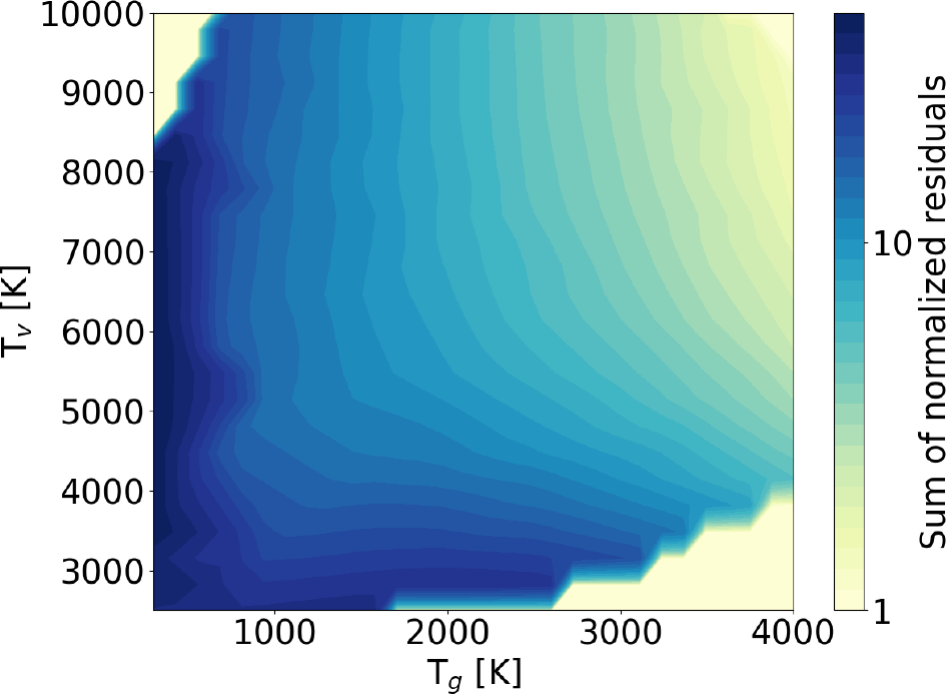
Map of the sum of normalized residuals of the FP solution with respect to the STS one, for a vibrational kinetic scheme including V–V_1_ and V–T processes, without dissociation.

**Figure 4:**
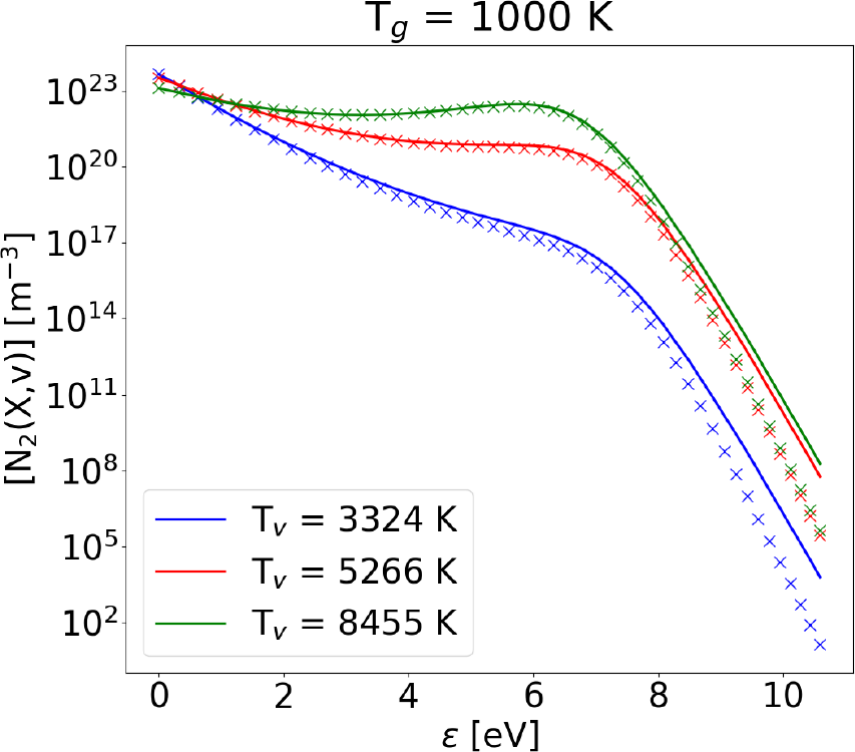
Vibrational distribution functions obtained with the FP approach (solid lines) compared to the ones obtained with the STS method (crosses) including V–V_1_ and V–T processes, without dissociation, at fixed gas temperature *T*_g_ = 1000 K.

As a proof of criteria (45) and (47), Figure 3 shows the sum of normalized residuals as a function of input *T*_g_ and *T*_*v*_ for vibrational kinetic schemes including both V–V_1_ and V–T processes, without dissociation. This quantity is calculated as
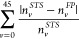
48where *n*_*v*_ is the population of vibrational level *v* and the superscripts refer to the method used to calculate it. Note that in this case, the STS code includes also monoquantum e–V collisions with the first two vibrational levels: this allows one to maintain the total number density constant and equal to the gas density, without having to impose external normalization; *n*_0_ and *n*_1_ obtained from the STS solution are then used as input for the model including the FP approach. This explains why the values of vibrational temperature shown in Figure 4 and 6 are not the same as in the previous subsection.

The general trend of improving agreement with increasing temperatures is highlighted in Figure 3. By following a line at a fixed vibrational temperature, the gradual improvement of the agreement with increasing gas temperature can be observed; the same is not true if *T*_g_ is fixed and *T*_*v*_ is increased (*n*_*e*_ is kept constant at 10^13^ cm^–3^, while the electron mean energy is varied from 0.5 to 2 eV). This is due to the fact that the most relevant contribution to the total sum of residuals is given by the tail of the distribution, where V–T processes, which are highly dependent on gas temperature, dominate. As an example of this, Figure 4 shows the results of the calculations of the VDF at fixed *T*_g_ and increasing vibrational temperature: while the bulk of the distribution is always in very good agreement with the STS solution, the tail is always overestimated by the Fokker–Planck approach. It is however worth noting that the knee of the distribution is always very well placed; since this feature marks the energy value where *J*_*V*–*V*1_(ϵ) = *J*_*V*–*T*_(ϵ), we can conclude that the Fokker–Planck formulation approximates very well the relative strength of V–V_1_ and V–T processes.

### V–V_*n*_

**Figure 5:**
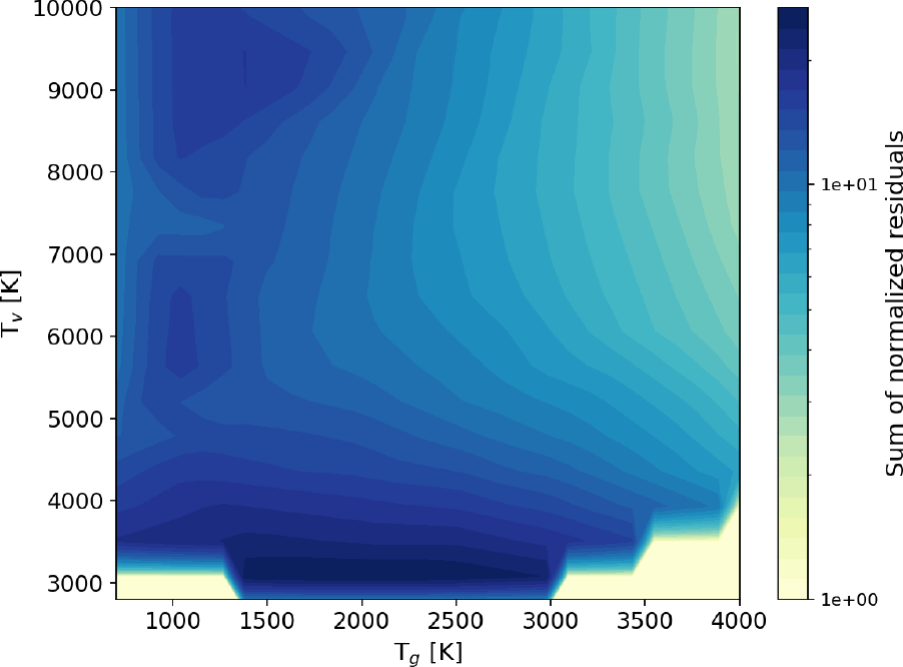
Map of the sum of normalized residuals of the FP solution with respect to the STS one, for a vibrational kinetic scheme including V–V_1_, V–T, and V–V_*n*_ processes, without dissociation.

The equilibrium distribution in the presence of only quasiresonant V–V transitions cannot be calculated analytically; therefore, it is not possible to conduct the analysis previously employed for V–V_1_ and V–T. Instead, an idea of the validity of the approximation can be given by plotting the sum of residuals over the temperature space, as shown in Figure 5, where FP and STS solutions including V–V_1_, V–T, and V–V_*n*_ (without dissociation) are compared. For *T*_g_ > 1000 K, it has been found that when virtually traversing the parameter space along a constant *T*_g_ line, the sum of normalized residuals reaches a minimum and increases as the degree of nonequilibrium is further increased. That trend was not present in the previous section, where quasiresonant processes were not included, and therefore points at V–V_*n*_ as its possible cause.

**Figure 6:**
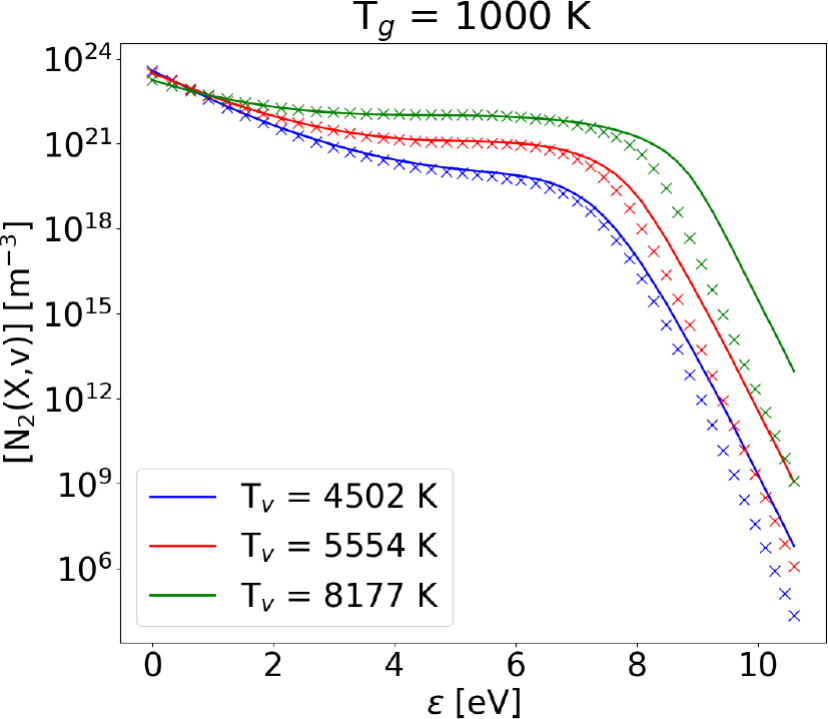
Vibrational distribution functions obtained with the FP approach (solid lines) compared to the ones obtained with the STS approach (crosses) including V–V_1_, V–T, and V–V_*n*_ processes, without dissociation, at a fixed gas temperature *T*_g_ = 1000 K.

As shown in Figure 6, the plateau of the distribution, which is the signature of V–V_*n*_ processes, is well captured by the STS solutions, while the FP solution tends to overestimate the population at the tail of the VDF and the length of the plateau. The misplacement of the knee of the distribution, which was not observed previously, is due to an overestimation of the contribution of V–V_*n*_ processes to the total flux in energy space, which in turns also causes a more severe overpopulation of the tail than what was already observed as an effect of the poorly approximated ratio between *A*_*V*–*T*_ and *B*_*V*–*T*_. This effect depends on the degree of nonequilibrium, rather than on the actual value of *T*_*v*_: for *T*_*v*_/*T*_g_ > 6, a more significant deterioration of the agreement with respect to the cases without V–V_*n*_ can usually be seen. This behavior gradually smooths out when *T*_g_ is increased and at 4000 K the residual map resembles the one in Figure 3, due to the dominance of V–T processes over V–V_*n*_.

The effect of the assumption made in eq 22 has been studied using as input values *T*_g_ = 1000 K and *T*_*v*_ = 8177 K in order to maintain a regime of high excitation, where V–V_*n*_ processes are relevant. In this case, the simulation was carried out for a fixed time of 10 ms, in order to allow comparison of the CPU times. The VDF is not changed by the use of the approximation in eq 22 in the FP approach. However, by avoiding the calculation of the two derivatives, the CPU time (around 20 s) is reduced by ∼20%.

### e–V and Vibrational Temperature

The presence of collisions between N_2_ molecules and electrons introduces the problem of determining the vibrational temperature self-consistently. As already explained in the Methods, three different methods are here compared: (i) the solution of a vibrational energy equation (eq 31), (ii) the implementation of a reduced STS approach for the population of N_2_(*v* = 0) and N_2_(*v* = 1), and (iii) a self-consistent temporal evolution of the FP solution. Accuracy and computational speed of the three methods are compared at three different values of electron density (10^11^ cm^–3^, 10^12^ cm^–3^, and 10^13^ cm^–3^) and varying the electron mean energy (ϵ_*e*_) between 1.2 and 2 eV. The kinetic scheme in this case includes monoquantum and multiquanta e–V, V–V_1_, V–T and V–V_*n*_ processes, without dissociation. The accuracy of each method is evaluated by calculating the sum of normalized residuals with respect to the STS solution.

**Figure 7:**
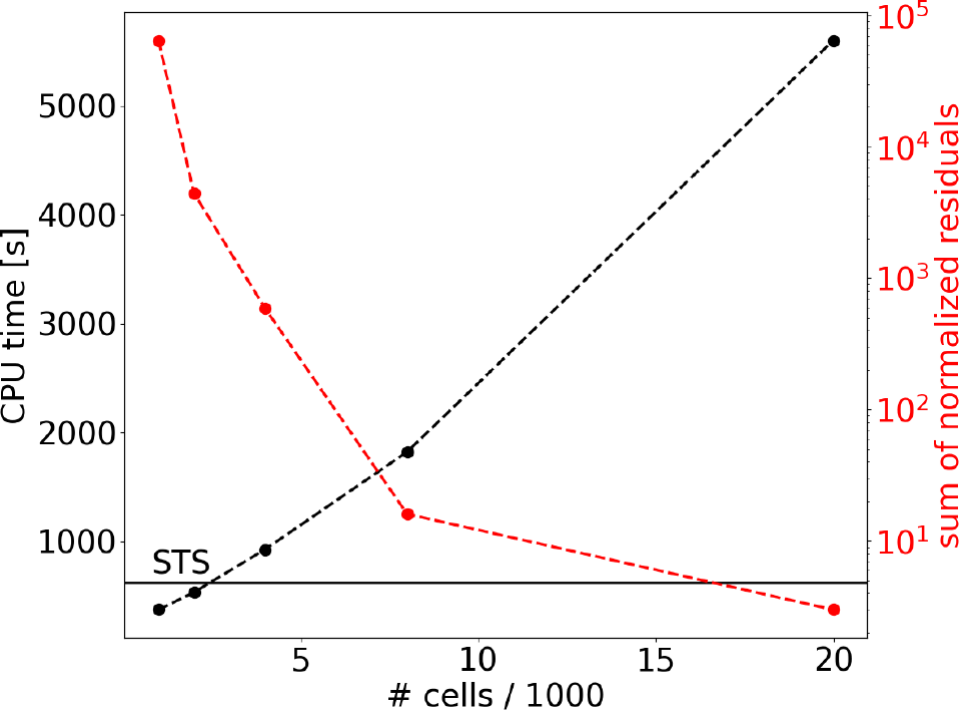
CPU time and sum of normalized residuals as a function of the number of cells, using *n*_*e*_ = 10^11^ cm^–3^ and ϵ_*e*_ = 1.4 eV, for the solution including FP only. The solid line marks the STS CPU time.

The self-consistent solution of the FP equation (method iii), without any additional calculation of the vibrational temperature, converges to the correct *T*_*v*_ and VDF if the number of cells is significantly increased. Figure 7 shows the effect of increasing the number of cells in which the energy space is divided on both the accuracy and the computational efficiency of the solution. The accuracy of the solution converges to a minimum value of the sum of normalized residuals of ∼1 when 20000 cells are used. The increased CPU time is mainly due to the increased number of drift and diffusion coefficients that are calculated at every iteration. Increase in the number of cells does not produce relevant changes in the solution if the other two methods are used, as *T*_*v*_ is estimated more accurately and is not subject to the values of *A* and *B*, which result from an approximation.

**Figure 8:**
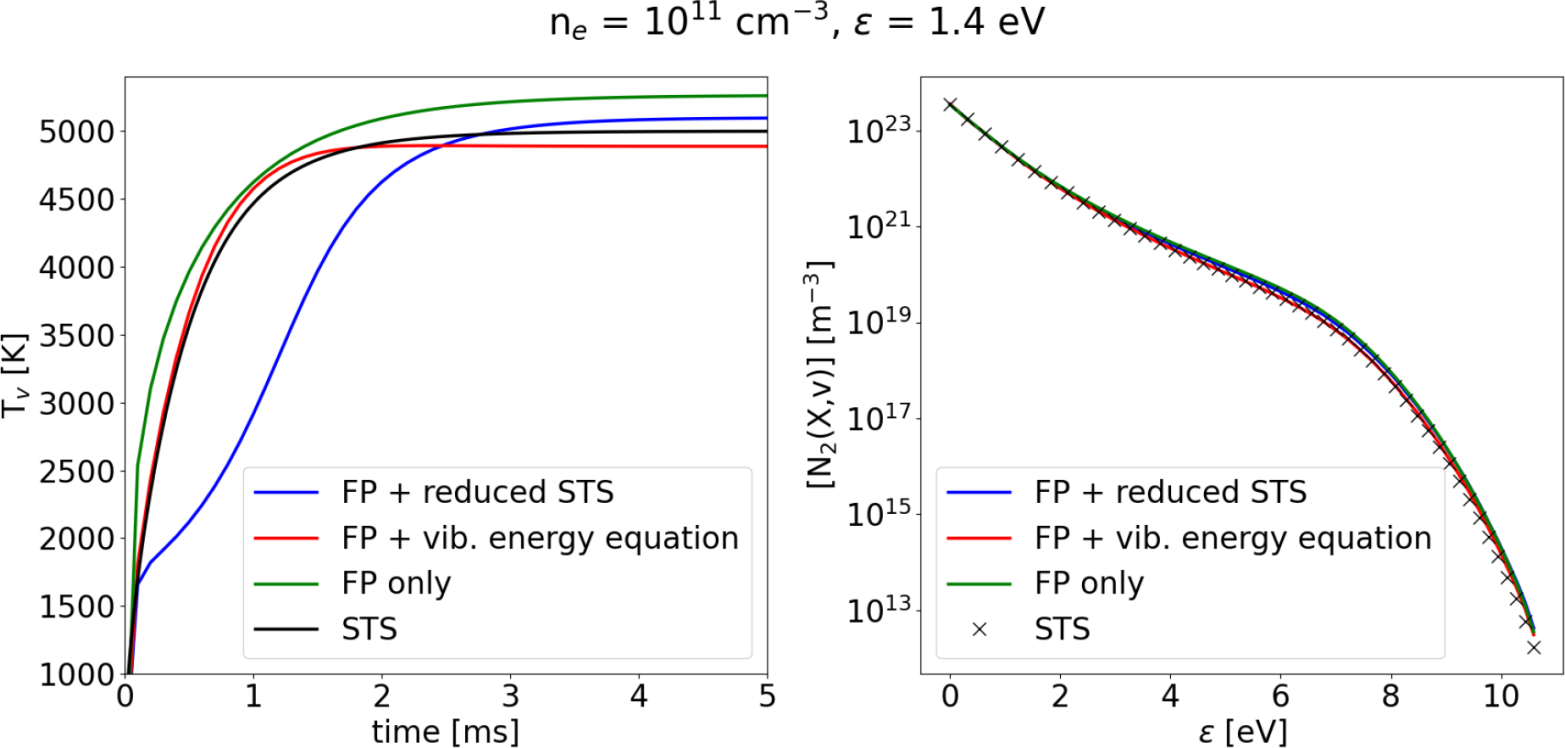
Evolution of *T*_*v*_ and final solution of the VDF obtained applying the three proposed methods, compared to the STS solution. Gas temperature is fixed at *T*_g_ = 1000 K.

Figure 8 shows the comparison between the three different methods both in terms of temporal evolution of the vibrational temperature and accuracy of the final steady state VDF. While the steady state VDF is reproduced remarkably well by all three methods, the time evolution of the vibrational temperature as obtained through the reduced STS approach diverges from the STS solution considerably during the first ∼2 ms.

The solutions obtained with the coupled vibrational energy equation and the reduced STS approach have comparable computational efficiency, employing ∼350 s of CPU time to simulate 10 ms of physical time, which is ∼40% more efficient than the STS calculation. The third approach, on the other hand, due to the increased number of cells, takes more than 10 times the STS CPU time to complete the simulation.

Most of the cost reduction in the solution of FP equation is related to the integration of the equation, which suggests its dependence on the solver used to perform such operation. For this reason, the simulation carried out in the previous section has been compared to solutions employing, instead of RADAU5, LSODA or forward method to solve the ME. This analysis revealed similar computational costs for forward, LSODE and FP approach with TDMA.

Most of the computational time in the solution of vibrational kinetics using FP approach is employed in the calculation of the drift and diffusion coefficients (A and B), which explains the ramping up in computational cost with the increased amount of cells in energy space. This process could be further optimized by providing parametric expressions of said coefficients with respect to relevant physical variables, similarly to what is done for such coefficients when movement in space is described.

The coupled vibrational energy equation is chosen for the self-consistent calculation of vibrational temperature, since it provides the best agreement in the temporal evolution of *T*_*v*_ and the best CPU time.

**Figure 9:**
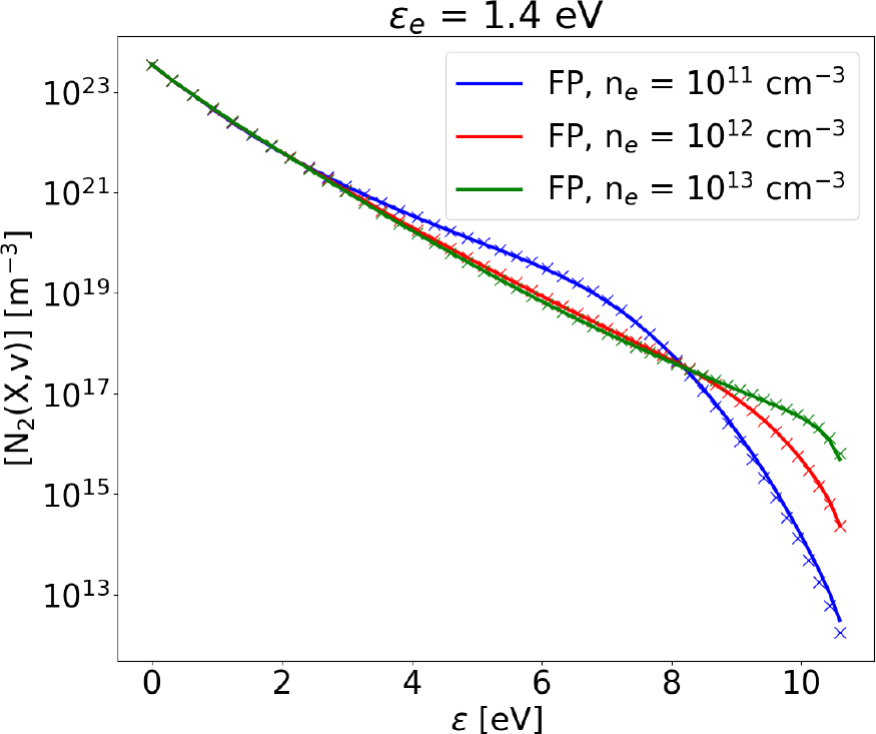
Steady state VDF calculated solving the FP equation (solid lines) compared to the STS solution (crosses), at ϵ_*e*_ = 1.4 eV and varying electron density. Gas temperature is kept fixed at *T*_g_ = 1000 K. The kinetic scheme in this case includes monoquantum and multiquanta e–V, V–V_1_, V–T, and V–V_*n*_ processes, without dissociation.

Finally, Figure 9 shows the comparison between FP and STS solutions for the VDF at varying electron density, using the vibrational energy equation for the calculation of *T*_*v*_. The agreement between the solutions is excellent.

**Figure 10:**
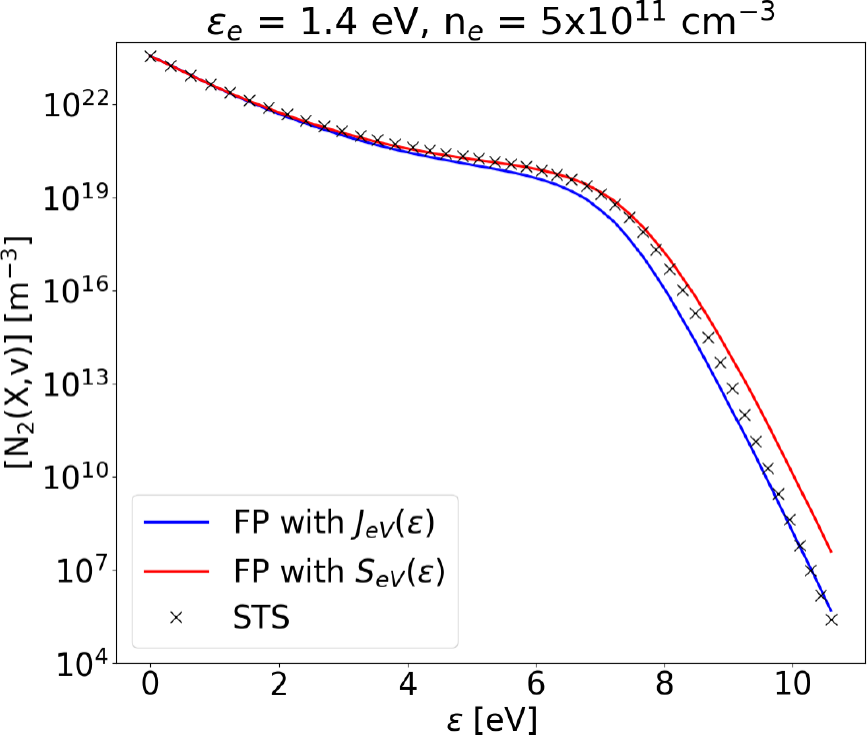
Steady state VDF obtained using the FP approach with *S*_*eV*_(ϵ) or *J*_*eV*_(ϵ), compared to the STS solution at ϵ_*e*_ = 1.4 eV, *n*_*e*_ = 5 × 10^11^ cm^–3^, and *T*_g_ = 1000 K. Dissociation is not included.

**Figure 11:**
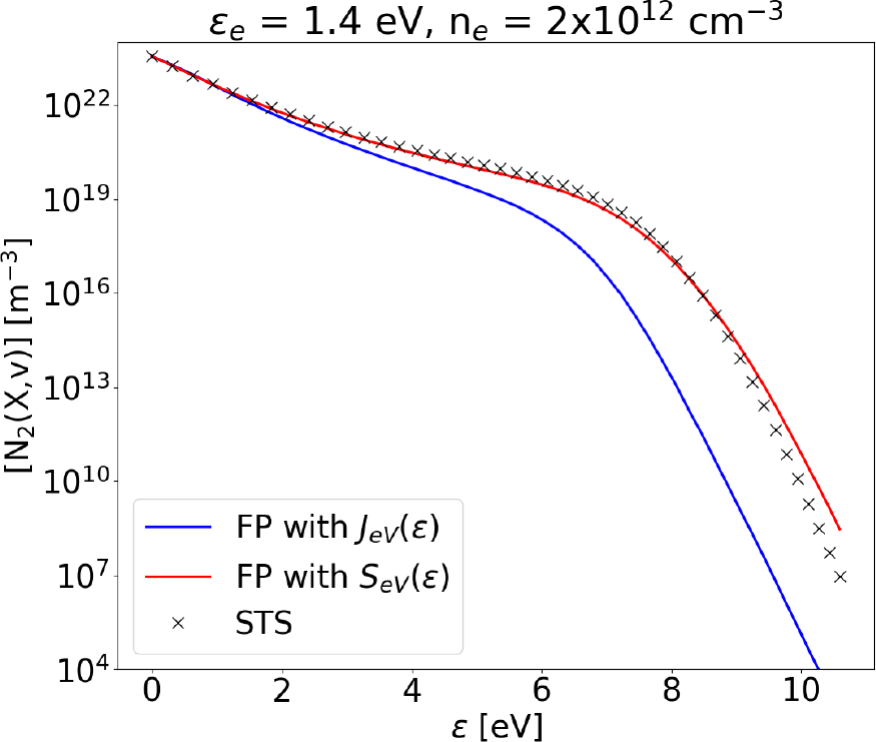
Steady state VDF obtained using FP approach with *S*_*eV*_(ϵ) or *J*_*eV*_(ϵ), compared to the STS solution at ϵ_*e*_ = 1.4 eV, *n*_*e*_ = 2 × 10^12^ cm^–3^, and *T*_g_ = 1000 K. Dissociation is not included.

The tests presented above are run by implementing e–V collisions as a source term, modifying the form of the FP eq (eq 8). However, as mentioned in the Methods, electron-driven vibrational excitation can also be modeled using the FP formulation (eq 2), by introducing *A*_*eV*_ and *B*_*eV*_. Due to the constraint on small energy jumps, the accuracy of that approach is higher for monoquantum transitions. In Figures 10 and 11, a kinetic scheme with only monoquantum e–V exchanges is considered and the solution of the STS code is compared to the one obtained by implementing *S*_*eV*_(ϵ) or *J*_*eV*_(ϵ).

The accuracy of the FP formulation for monoquantum e–V is higher at low electron density, where V–V relaxation is faster (*k*_*V*–*V*1_(ϵ)*n*_1_ > *k*_*eV*_(ϵ)*n*_*e*_) (Figure 10). On the other hand, if the electron density is increased to ∼2 × 10^12^ cm^–3^ (Figure 11), the formulation introducing *J*_*eV*_ breaks down. In particular, the effect of e–V collisions on intermediate and higher levels is severely overestimated, meaning that the flux in energy space *J*_*eV*_(ϵ) is dominating on , which is responsible for populating intermediate levels; this causes an evident decrease in their population, as e–V collisions try to push the VDF to a Boltzmann distribution. Note that *n*_*e*_, as shown in eq 29 and 30, does not modify the shape or the ratio of *A*_*eV*_ and *B*_*eV*_ but only their value relative to other processes. The sum of normalized residuals for the method implementing the source term *S*_*eV*_(ϵ) remains constant for increasing electron density, while such sum increases from ∼10^2^ to ∼10^13^ as electron density is increasing from 5 × 10^11^ cm^–3^ to 2.5 × 10^12^ cm^–3^ if *A*_*eV*_ and *B*_*eV*_ are used, instead. It should be noted that, when increasing the mean electron energy (ϵ_*e*_ > 3 eV), both methods reach a very good agreement.

### V–V_2_

**Figure 12:**
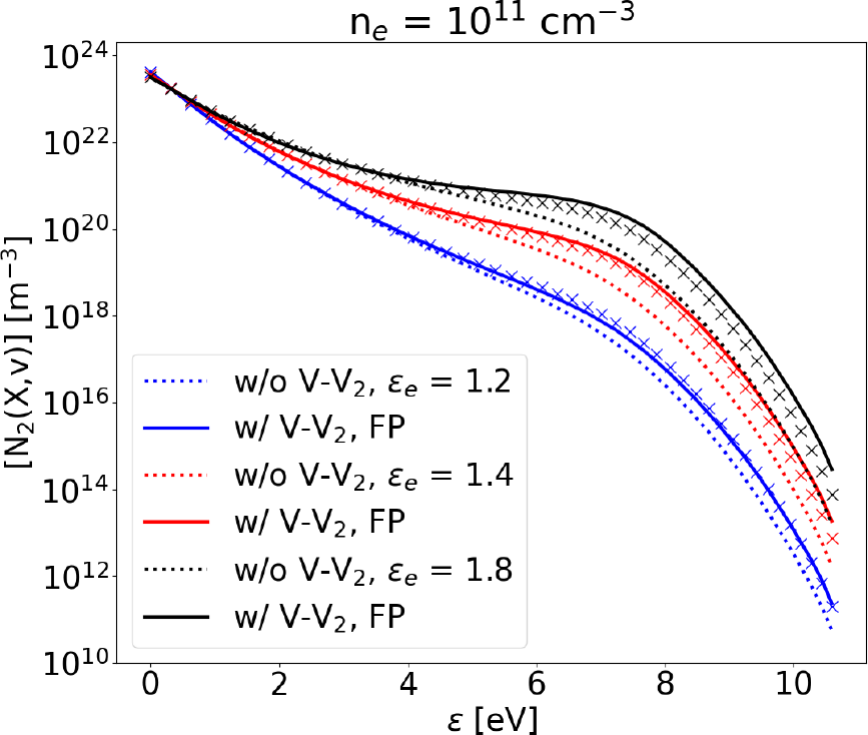
Steady state VDF calculated solving the FP equation (solid lines) compared to the STS solution with (crosses) and without (dotted lines) V–V_2_, at different values of electron mean energy, *n*_*e*_ = 10^11^ cm^–3^ and *T*_g_ = 1000 K. Dissociation is not included.

**Figure 13:**
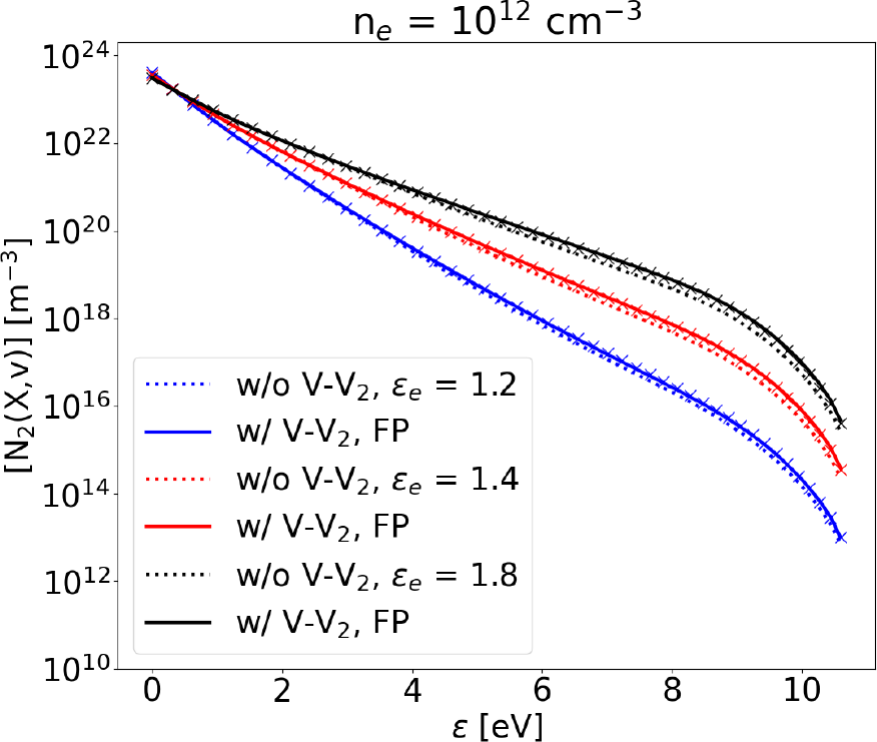
Steady state VDF calculated solving the FP equation (solid lines) compared to the STS solution with (crosses) and without (dotted lines) V–V_2_, at different values of electron mean energy, *n*_*e*_ = 10^12^ cm^–3^ and *T*_g_ = 1000 K. Dissociation is not included.

The effect of additional nonresonant V–V collisions has been investigated by adding V–V_2_ processes to the vibrational kinetics scheme. At low electron density the effect is clear in the tail of the distribution, which is slightly more populated with respect to the case omitting V–V_2_ (Figure 12). As electron density is increased by 1 order of magnitude (Figure 13), the effect of V–V_2_ collisions becomes less evident, due to the dominance of e–V processes. Nonresonant V–V_*i*_ collisions with molecules in vibrational level *i* > 2 are progressively less probable as *i* increases, due to the progressively lower density of such levels. For this reason, we decide to run our simulations without those processes that have *i* > 2.

Note that both Figure 12 and 13 show the very good agreement between the solution obtained with the STS approach and the FP one, opening the possibility of adding further nonresonant V–V processes, if needed.

### Collisions with Atomic Nitrogen

**Figure 14:**
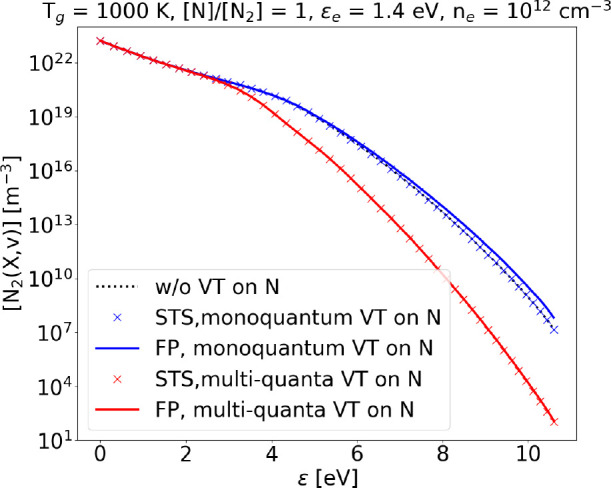
Steady state VDF obtained with FP and STS approach at *T*_g_ = 1000 K, ϵ_*e*_ = 1.4 eV, and *n*_*e*_ = 10^12^ cm^–3^, considering only monoquantum or also multiquanta V–T collisions.

**Figure 15:**
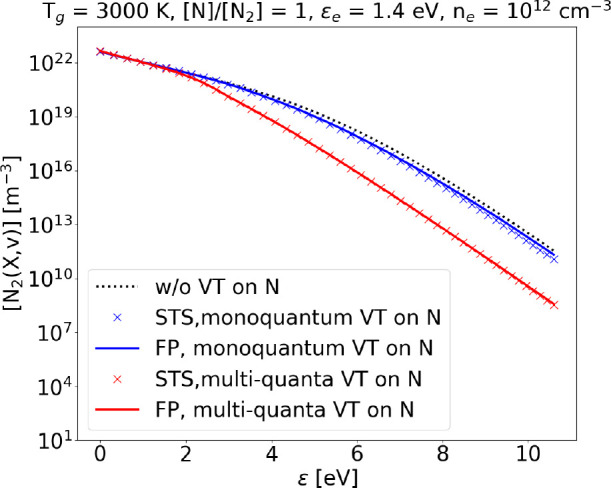
Steady state VDF obtained with FP and STS approach at *T*_g_ = 3000 K, ϵ_*e*_ = 1.4 eV, and *n*_*e*_ = 10^12^ cm^–3^, considering only monoquantum or also multiquanta V–T collisions.

As the expression for drift and diffusion coefficients for monoquantum V–T exchanges between vibrationally excited N_2_ and nitrogen atoms is formally equal to the one given for V–T collisions with N_2_, the same limits of validity of the FP approach stand, namely the condition *T*_g_ > Δ*E*(ϵ). In order for V–T,N collisions to visibly modify the steady state VDF, the dissociation fraction has been fixed at 50%, along with constant mean electron energy (ϵ_*e*_ = 1.4 eV) and electron density (*n*_*e*_ = 10^12^ cm^–3^). In order to study the effect of collisions with atomic nitrogen on the VDF, the included reactions for vibrational kinetics are the ones listed in Table 1. First, results obtained by implementing exclusively monoquantum V–T collisions with N have been compared to the STS solution, with the aim of assessing the accuracy of the description of such processes in a FP framework using a flux formulation. Figures 14 and 15 reveal that V–T collisions causing the loss of a single quantum of energy do not modify relevantly the vibrational distribution function; the dotted line in both figures is the VDF obtained without processes involving atomic nitrogen, calculated using the STS approach. Attempts at higher dissociation fraction yield similar results. A slight depopulation of the tail of the distribution is seen at *T*_g_ = 3000 K, due to the increased rate coefficient of collisions with atomic nitrogen. Collisions involving the exchange of more than one quantum introduce a significant depopulation of the high energy vibrational levels. The FP approach, implementing multiquanta transitions in the form of a source term, reproduces the VDF resulting from the STS model.

### Dissociation Mechanisms

Dissociation mechanisms are added to the vibrational kinetics. In order to isolate the effect of dissociation, electron mean energy and electron density have been kept fixed at ϵ_*e*_ = 1.4 eV and *n*_*e*_ = 10^12^ cm^–3^, respectively.

**Figure 16:**
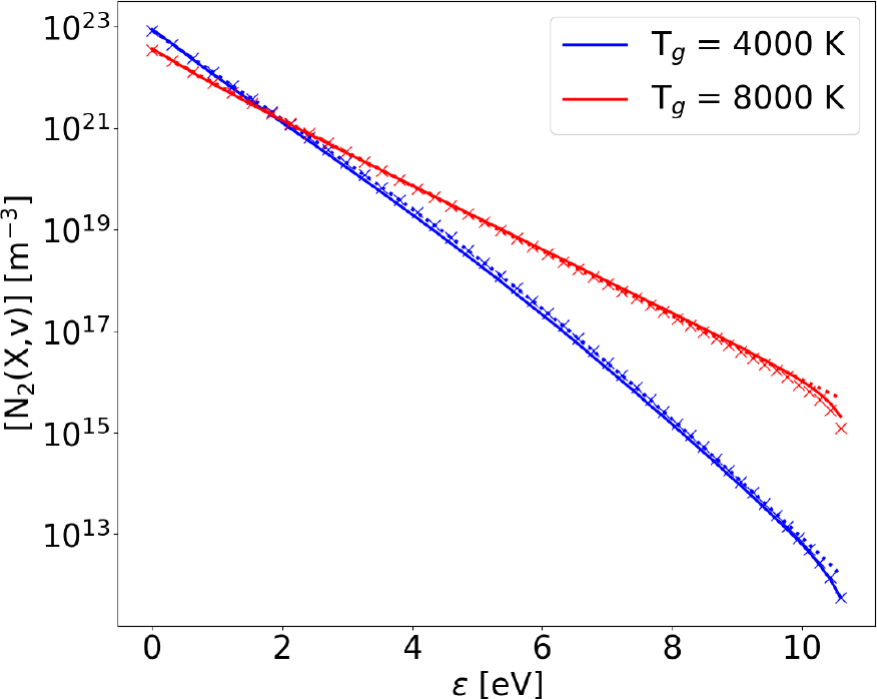
Steady state VDF obtained with FP (solid lines) and STS (crosses) approach at ϵ_*e*_ = 1.4 eV, *n*_*e*_ = 10^12^ cm^–3^, including all reactions in Table 1, compared to the case excluding dissociation (dotted lines).

Figure 16 shows the comparison between FP and STS steady state solutions of the vibrational kinetics detailed in Table 1, including also the STS solution for the case without dissociation. The choice of temperature was dictated by the absence of a relevant difference between the case with and without dissociation at lower temperatures. In fact, at lower *T*_g_, lower values of *T*_*v*_ are found, causing a lower population of the levels involved in the dissociation processes listed in Table 1 and therefore decreasing their rate.

The main effect of dissociation is the decrease in the population of the very last level; this is due to the absence of a recombination process causing the formation of N_2_(*v* = 45). Adding a reverse reaction:^51^
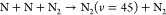
49with the rate coefficient calculated with detailed balance would lead to the absence of the fall in the tail.

### Time Evolution

Finally, the time-resolved results obtained from the STS code, including all processes in Table 1 have been compared to the results obtained using the FP approach to vibrational kinetics, under the same physical and numerical conditions. In particular, the gas temperature and the time step have been kept constant at 1000 K and 10^–8^ s, respectively. Electron density and electron mean energy have been kept constant at 10^12^ cm^–3^ and 1.4 eV, respectively, for 1 ms of physical time and reduced to 10^4^ cm^–3^ and 0.03 eV for the rest of the simulation.

**Figure 17:**
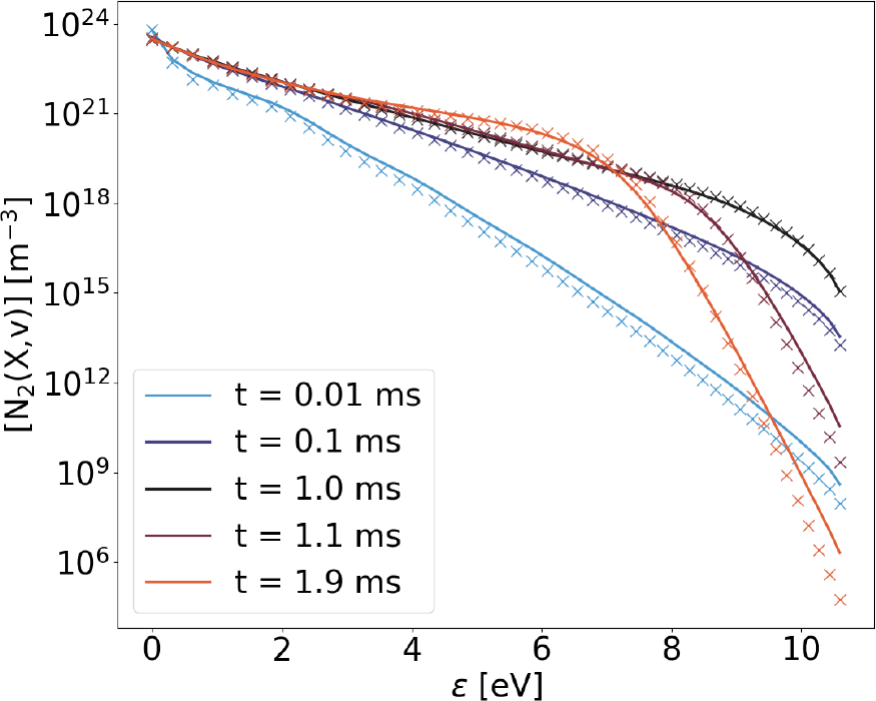
VDFs obtained with FP (solid lines) and STS (crosses) approach at different times (*t*) during the simulated 2 ms, including all reactions in Table 1. Input parameters are discussed in the text.

As shown by Figure 17, very good agreement with the STS solution is achieved at all the chosen values of time (*t*), both before (*t* ≤ 1 ms) and after (*t* > 1 ms) the removal of electrons from the simulation.

## Conclusions

In this work, the Fokker–Planck approach to vibrational kinetics was extended to allow a complete description of vibrational kinetics. A novel model based on the extended FP approach has been developed. The FP equation is solved in a discretized energy grid using the finite volume technique. Results of the new model have been benchmarked against STS ones for the case of vibrational kinetics in N_2_. The implementation of each process on the FP model has been demonstrated, together with its influence on vibrational kinetics. This includes multiquanta transitions due to both V–T and e–V collisions, which have been modeled through additional source terms in the FP equation.

The steady state VDFs obtained with the FP approach show very good agreement with the STS solutions. Comparisons over a wide range of input parameters have revealed limits of validity of the model. In particular, V–V_1_ and V–T relaxation have been shown to be accurately modeled for *T*_*v*_ > 3700 K and *T*_g_ > 1700K, and for *T*_g_ > 3700 K, respectively. The addition of quasiresonant V–V collisions yields accurate results, provided that the degree of nonequilibrium *T*_*v*_/*T*_g_ is lower than 6. It has to be noted that if e–V and superelastic collisions are implemented, the agreement with the STS solution is excellent over a wide range of electron densities and mean energies.

Moreover, three different methods for the self-consistent temporally resolved calculation of vibrational temperature have been compared. The implementation of a vibrational energy balance equation proved to be the most accurate and computationally efficient method among the three. The solution of a stand-alone FP equation showed remarkably good agreement with the STS solution, but only with a number of control volumes more than 20 times higher than the other methods and a consequent significantly lower computational efficiency.

This new implementation of the FP method allowed us to study in details the limitation of the FP approach and to benchmark it with STS results also including multiquanta transitions and time-dependent solutions, that, as already stated, were not possible with the flux-matching approach that was adopted previously.

In terms of computational efficiency the FP approach allows to scale the CPU time by a factor 0.6, compared to a model implementing a robust Runge–Kutta ODE solver, while results in the same CPU time employed by LSODA and by a simple forward solver. This gain in computational efficiency with respect of a Runge–Kutta solver is lower than in previous works employing the flux-matching approach, but the new model offers more versatility, with the possibility of temporal resolution and multiquanta transitions. Moreover, the nature of the description of the problem offers the possibility to parametrize A and B coefficients as a function of relevant physical parameters, like vibrational or gas temperature, instead of calculating them from rate coefficients, allowing further cutting of computational time.

Further development of the code to make it computationally competitive are required; based on the current analysis, such efforts must focus on improvement of the calculation of A and B coefficients. Backed by the excellent agreement between FP and STS solutions, the aforementioned parametrization of A and B as a function of relevant physical variables could offer intuitive and immediate insights into energy transport within the vibrational manifold, without having to disentangle the hundreds of rate coefficients used otherwise. Additionally, computational time could be saved by creating a nonuniform mesh in energy space, which is straightforward with the fluid-like description.

Moreover, treatment of the vibrational manifold as a continuum makes the approach easily adaptable to the case of 61 vibrational levels of nitrogen, since no additional pseudospecies need to be added, as opposed to the STS approach. Changes in vibrational kinetics due to the modified description of the N_2_ molecule could also be readily detected by simply looking at drift and diffusion coefficients in energy space.
